# Treatment patterns and healthcare resource utilization in pediatric neurofibromatosis type 1-associated symptomatic plexiform neurofibroma in the United States from 2000 to 2019

**DOI:** 10.1093/nop/npag038

**Published:** 2026-06-13

**Authors:** Surya P Rednam, Theresa Dettling, Michael Blackowicz, Randolph de la Rosa Rodriguez, Xiaoqin Yang, Saima Ali, Julia Meade

**Affiliations:** Texas Children’s Hospital, Baylor College of Medicine, Houston, Texas, USA; Alexion, AstraZeneca Rare Disease, Boston, Massachusetts, USA; Alexion, AstraZeneca Rare Disease, Boston, Massachusetts, USA; Alexion, AstraZeneca Rare Disease, Boston, Massachusetts, USA; Value and Implementation Outcomes Research, Merck & Co., Inc., Rahway, New Jersey, USA; Texas Children’s Hospital, Baylor College of Medicine, Houston, Texas, USA; Department of Pediatrics, University of Pittsburgh School of Medicine, Pittsburgh, Pennsylvania, USA (J.M.)

**Keywords:** healthcare resource utilization, neurofibroma, neurofibromatosis type 1, plexiform neurofibromas

## Abstract

**Background:**

Neurofibromatosis type 1 (NF1) is a genetic condition that affects an estimated 1 in 3000 people worldwide. Between 30% and 50% of patients with NF1 develop symptomatic, inoperable plexiform neurofibromas. To identify the unmet needs and understand the potential value of new treatments, the treatment patterns, healthcare resource utilization (HCRU), and clinical outcomes were evaluated in a real-world cohort of 102 pediatric patients with NF1 and PN.

**Methods:**

Retrospective data were collected from patients diagnosed with NF1 and symptomatic, inoperable PNs from 2000 to 2018 across 3 US sites. Data on clinical characteristics, site visits, treatments, and outcomes were collected through 2019.

**Results:**

Patients with NF1, on average, developed 3.5 symptomatic, inoperable PNs during the study. PN complications included pain (76.5%), disfigurement (45.1%), and motor dysfunction (13.7%). During the study period, which was prior to Food and Drug Administration (FDA) approval of mitogen-activated protein kinase inhibitors for NF1, available treatments attempted to address the symptoms caused by PNs but did not typically slow or prevent their growth. Surgery was performed for 38.1% of study patients. At follow-up of ≥12 months, 62.5% of PNs regrew after surgery. Each year, patients made, on average, 6 visits to an outpatient care center and 1 visit to an oncologist. Nearly all patients (99.0%) received at least 1 diagnostic imaging scan, with 3.4 scans per year on average.

**Conclusions:**

This study describes the high burden of HCRU among pediatric patients with NF1-PN prior to FDA approval of pharmacologic therapies for PN and demonstrates the need for effective treatment options.

Key Points

- Between 30% and 50% of patients with neurofibromatosis type 1 (NF1) develop plexiform neurofibromas (PNs).
- PNs can cause pain, disfigurement, and motor dysfunction.
- Prior to MEK inhibitors, medical treatment for PN often addressed only symptoms.

Importance of the Study

Plexiform neurofibromas (PNs) are benign tumors associated with neurofibromatosis type 1 (NF1) that can grow rapidly during childhood, requiring intervention to mitigate further debilitating complications. Few studies have characterized the clinical, social, and economic burden of NF1-PN. Developing a descriptive picture of NF1 and associated PNs across a large patient population can address critical information gaps in the characterization of clinical features, treatment selection, and outcomes in NF1, which is necessary to identify unmet need and assist in understanding the potential value of new therapeutic approaches.

Neurofibromatosis type 1 (NF1), an autosomal, dominant genetic condition, is characterized by cutaneous hyperpigmented lesions (café-au-lait macules and intertriginous freckling), as well as growth of benign tumors on or under the skin (cutaneous neurofibroma) and potentially deeper and/or larger masses along nerves within the body (plexiform neurofibromas [PNs]).^1^ Features of NF1 manifest in infancy with a progression of expected features and the emergence of risks as affected individuals age, including disfigurement, vision loss, and the development of cancer.^1^^,^^2^ Neurofibromatosis type 1 affects an estimated 1 in 3000 people worldwide.^1^ Significant phenotypic variability exists in NF1. This includes the presence, absence, and severity of a variety of NF1-associated features: neurofibroma burden; development of low-grade glioma involving the central nervous system, particularly involving the optic pathway; neurocognitive, neurodevelopmental, and neurobehavioral concerns; vasculopathy-related issues; endocrinopathies; and skeletal conditions.^3^^,^^4^ Approximately 30%-50% of individuals with NF1 develop PNs.^5–7^ Over time, PNs may progress, causing pain, functional disability, or disfigurement, or they may even become life-threatening if the tumors compress vital structures or undergo transformation to malignant peripheral nerve sheath tumors.^5–8^ For people with NF1, the progressive impact of the condition on appearance, functional status, pain, and physical and mental health, particularly among patients who develop PNs, significantly reduces quality of life.^9^^,^^10^

Plexiform neurofibromas may rapidly grow in childhood, often outpacing increases in body size over time, and while PNs can occur anywhere in or on the body, more than 60% of symptomatic PNs in children occur on the head and neck.^6^^,^^11^^,^^12^ For the significant proportion of PNs that progress, patients may undergo periodic MRI to objectively assess the size of PNs and the effect of PNs on important local structures (eg neck vessels, spinal cord).^12^ Surgery may be used to mitigate the development of debilitating complications.^12–14^ However, PNs often cannot be completely excised due to size or proximity to vital tissues, and effective drug therapies are limited.^6^ Selumetinib (ARRY-142886, AZD6244), a mitogen-activated protein kinase 1/2 (MEK1/2) inhibitor, was approved by the United States (US) Food and Drug Administration (FDA) for the treatment of pediatric patients (ages >1) with NF1 and symptomatic, inoperable PNs (NF1-PN) in capsule formulation in 2020 and in oral granule formation in 2025 for patients with difficulty swallowing capsules.^15^ Prior to selumetinib’s approval, treatment for these patients was largely limited to approaches for managing symptoms (eg controlling pain).^6^^,^^15–17^ In 2025, another MEK1/2 inhibitor, mirdametinib, was also approved by the FDA for this population.^18^

The understudied impact of NF1-PN on patients and their families needs further research to explore its clinical, social, and economic burdens. Unmet needs and treatment potential for this population can be better understood by characterizing the clinical features, treatment selection, and outcomes in NF1-PN. The aim of this study was to describe treatment patterns, healthcare resource utilization (HCRU), and clinical outcomes in a real-world cohort of pediatric patients with NF1-PN who had symptomatic, inoperable PNs in the US prior to FDA approval of MEK inhibitors.

## Methods

### Study Design

This study was a retrospective medical record review of pediatric patients with NF1 and symptomatic, inoperable PNs from 3 treatment centers across the United States with comprehensive NF1 clinics: Texas Children’s Hospital (Houston, TX), Children’s Hospital of Pittsburgh (Pittsburgh, PA), and Cincinnati Children’s Hospital Medical Center (Cincinnati, OH). Sites were selected due to their large patient populations, the feasibility of their being able to provide the data necessary to meet the study objectives, and their expertise and leadership in the treatment and management of NF1 and NF1-related symptomatic, inoperable PNs. Designated sites provided comprehensive patient care, including routine health supervision from multidisciplinary teams of expert medical providers for managing the broad spectrum of NF1-related manifestations, encompassing both medical and psychosocial needs. Patient records were abstracted if the patient received an NF1 diagnosis from January 1, 2000 to December 31, 2018; was aged 2-18 years at the time of diagnosis; was diagnosed with ≥1 symptomatic, inoperable PN; received onsite care for ≥12 months; and had ≥3 institutional medical visits prior to the end of the study period on December 31, 2019. These records were abstracted at the patient’s first, last, and “midpoint” visits (the visit nearest the midpoint between the first and last). Abstracting physicians were asked to select diagnostic criteria for and morbidities and manifestations of NF1 from a list of options determined by literature searches and consultation with clinicians. Abstracting physicians were asked to select whether their patients had undergone treatments in certain categories (eg chemotherapy, radiotherapy, pharmacotherapy); definitions were not provided for these categories, and they were therefore open to physician determination. Classes of medications (eg analgesics) were assigned according to definitions in the United States Pharmacopeia.^19^ This study was reviewed and approved by site-specific institutional review boards as well as by the RTI International Institutional Review Board.

### Data Analysis

Over the course of the study period, data were collected on the characteristics of the patients with NF1-PN at each site, the number of site visits, clinical characteristics, PN-related treatment and monitoring characteristics, and patient outcomes. Some variables, such as the number of years between birth and diagnosis, time between diagnosis of NF1 and initial diagnosis of PNs, and the duration of site visits, were calculated from patient records. All study measures were described through tabular display of means, medians, ranges, and SDs (indicated by ±) for continuous variables and frequency distribution (number and percentage) for categorical variables, as appropriate. For HCRU questions, all measures were presented over available follow-up time and annualized (to account for differing lengths of follow-up time across patients) and were described overall and by care setting/type. HCRU items included frequency of healthcare visits (ie inpatient, outpatient, emergency department, primary care, and specialty care settings), length of stay, as well as diagnostic imaging. All analyses were descriptive in nature and were conducted using SAS Studio statistical software (version 3.8) (SAS Institute, Inc.).

## Results

### Demographic and Clinical Characteristics

A total of 102 pediatric patients with NF1 and ≥1 symptomatic, inoperable PNs were included from the 3 participating sites, with a mean treatment time of 8.3 years (Table 1). Patients had a median of 20 visits, with most patients (62.7%) having more than 15 visits during the study observation period. Most patients were non-Hispanic (72.5%), White (71.6%), and male (64.7%). The median ages at NF1 diagnosis and at diagnosis of the first inoperable PN were 2 years and 6 years, respectively.

**Table 1.** Participant demographics and clinical characteristics.

Characteristic	Patients (*N* = 102)
Sex, *n* (%)	
Female	36 (35.3)
Male	66 (64.7)
Race, *n* (%) ^a^	
Asian, Native Hawaiian, Pacific Islander	2 (2.0)
Black/African American	22 (21.6)
Native American	1 (1.0)
White	73 (71.6)
Unknown	5 (4.9)
Ethnicity, *n* (%)	
Hispanic	24 (23.5)
Non-Hispanic	74 (72.5)
Unknown	4 (3.9)
Patients with age at NF1 diagnosis reported, *n* (%)	90 (88.2)
Age at NF1 diagnosis, years, *n* (%)	
≤2	59 (65.6)
3-6	20 (22.2)
7-10	8 (8.9)
11-13	1 (1.1)
≥14	2 (2.2)
Age at diagnosis of first symptomatic, inoperable PN, years, *n* (%)	
≤2	21 (20.6)
3-6	31 (30.4)
7-10	33 (32.4)
11-13	9 (8.8)
≥14	8 (7.8)
Other tumor diagnoses, *n* (%) ^a^	
Optic pathway gliomas	44 (43.1)
Cutaneous neurofibromas	32 (31.4)
Unresectable PN	18 (17.6)
Subcutaneous neurofibromas	13 (12.7)
Malignant peripheral nerve sheath tumor	4 (3.9)
Noncommon neurofibroma	1 (1.0)
Resectable PN	1 (1.0)
None/low-grade	27 (26.5)
Unknown	1.0 (1.0)
Morbidities and complications (history of and/or actively treated), *n* (%)^a^	
Any NF1 comorbidity	69 (67.6)
ADHD/ADD	46 (45.1)
Chronic constipation	18 (17.6)
Migraine	18 (17.6)
Hypertension	16 (15.7)
Cognitive disability	15 (14.7)
Depression	8 (7.8)
Epilepsy	5 (4.9)
Osteopenia	5 (4.9)

Abbreviations: ADD, attention-deficit disorder; ADHD, attention-deficit/hyperactivity disorder; NF1, neurofibromatosis type 1; PN, plexiform neurofibroma.

Data in this table was collected in the period during which the patient was seen in the clinic during the study period, between the first visit and the last visit (prior to December 31, 2019).

a Multiple responses allowed, so rows may add up to greater than 100%.

### NF1 Diagnosis and Disease Presentation

Most patients received their NF1 diagnosis (65.7%) and care (86.3%) from a geneticist (Table 2). The most common criteria to diagnose NF1 were the presence of 6 or more café-au-lait macules (87.3%), freckling in the axillary or inguinal regions/Crowe signs (45.1%), and ≥1 PN or 2 neurofibromas of any type (35.3%). At least 2 criteria must be met for clinical diagnosis of NF1, according to the National Institutes of Health Consensus Development Conference.^20^ Nearly all patients (94.1%) had at least 1 NF1-related manifestation (Table 2), including neuropathic pain (54.9%), behavioral deficits (42.2%), and migraines (39.2%). Additionally, attention-deficit/hyperactivity disorder or attention-deficit disorder (45.1%) and cognitive disabilities (14.7%) were common.

**Table 2.** NF1 diagnosis and characteristics.

	All patients (*N* = 102)
Criteria used to diagnose NF1 in selected patients (from the National Institutes of Health Consensus Development Conference), *n* (%) ^a^	
6 or more café-au-lait macules that are ≥5 mm in greatest diameter in prepubescent individuals and that are 15 mm in greatest diameter in postpubescent individuals	89 (87.3)
Freckling in the axillary or inguinal regions (Crowe sign)	46 (45.1)
2 or more neurofibromas of any type or 1 PN	36 (35.3)
First-degree relative (parent, sibling, or offspring) with NF1 by the above criteria	32 (31.4)
2 or more Lisch nodules (iris hamartomas)	20 (19.6)
Optic glioma	14 (13.7)
Other (positive genetic test)	9 (8.8)
Patient diagnosed elsewhere; criteria unknown	8 (7.8)
Distinctive osseous lesion^a^	3 (2.9)
Type of specialist that initially diagnosed the patient’s NF1, *n* (%) ^b^ ^,^ ^c^	
Geneticist	67 (65.7)
Pediatrician	19 (18.6)
Radiologist	18 (17.6)
Neurologist	10 (9.8)
Surgeon	10 (9.8)
Other	10 (9.8)
Pediatric neurologist	8 (7.8)
Dermatologist	3 (2.9)
Pediatric oncologist	3 (2.9)
Specialty of the physician primarily treating the patient’s NF1 at the clinic, *n* (%) ^b^	
Geneticist	88 (86.3)
Pediatric oncologist	36 (35.3)
Pediatric neurologist	26 (25.5)
Pediatric neuro-oncologist	24 (23.5)
Surgeon	24 (23.5)
Other	21 (20.6)
Neurologist	10 (9.8)
Neuro-oncologist	6 (5.9)
Pediatrician	6 (5.9)
Dermatologist	2 (2.0)
Oncologist	2 (2.0)
NF1-related manifestations, *n* (%) ^b^	
Any NF1 manifestation	96 (94.1)
Neuropathic pain	56 (54.9)
Behavioral deficits	43 (42.2)
Migraine, headache	40 (39.2)
Scoliosis and other skeletal deformities	39 (38.2)
Cosmetic concerns	31 (30.4)
Impairment of cognitive development	26 (25.5)
Difficulty with mobility	18 (17.6)
Limb-length inequality	18 (17.6)
Bone issues	17 (16.7)
High blood pressure/hypertension	16 (15.7)
Psychosocial issues	15 (14.7)
Small stature	12 (11.8)
Pectus deformity	7 (6.9)
Delayed or early puberty	6 (5.9)
Hemihypertrophy	6 (5.9)
None	6 (5.9)
Congenital tibial dysplasia	2 (2.0)
Microcephaly	0 (0.0)

Abbreviations: NF1, neurofibromatosis type 1; PN, plexiform neurofibroma.

a For example sphenoid wing dysplasia or thinning of long bone cortex, with or without pseudarthrosis.

b Multiple responses allowed, so rows may add up to greater than 100%.

c Some specialist types may have been reported differently at different clinical locations; for example, at Texas Children’s Hospital, there is no distinction between pediatric neuro-oncologists and neuro-oncologists.

### PN Characteristics, Complications, Progression, and Treatment

Clinical characteristics of PNs are presented in Table 3. Geneticists (68.6%) and radiologists (32.4%) most often initially diagnosed PNs. Pain (55.9%) and disfigurement (42.2%) were the most frequently reported signs and symptoms observed at the time of initial diagnosis of PNs. The median number of symptomatic, inoperable PNs per patient was 1.

**Table 3.** PN clinical characteristics and complications.

Characteristic	Patients (*N* = 102)
Mean number of PNs present at first encounter in the clinic ± SD	1.3 ± 1.0
Mean number of PNs developed while under surveillance ± SD	3.6 ± 9.8
PN complications, *n* (%) ^a^	
Pain	78 (76.5)
Disfigurement	46 (45.1)
Other ^b^	45 (44.1)
Motor dysfunction	14 (13.7)
Other organ/nerve compression	8 (7.8)
Sensory deficit	7 (6.9)
Vision loss	2 (2.0)
Spinal cord compression	1 (1.0)
None	1 (1.0)
Had ≥1 instance of disease progression (excluding death), *n* (%)	75 (73.5)
Symptoms associated with disease progression (among patients with ≥1 instance of progression, n (%) ^a^	
Pain	52 (69.3)
Disfigurement	42 (56.0)
Other^c^	35 (46.7)
Cognitive	13 (17.3)
Neurologic	10 (13.3)
Motor dysfunction	9 (12.0)
Methods of detection of disease progression, *n* (%) ^a^	
MRI	64 (85.3)
Clinical assessment without scans	41 (54.7)
Parent/patient report	24 (32.0)
PET scan	6 (8.0)
X-ray	6 (8.0)
Other	5 (6.7)
Laboratory tests	3 (4.0)
Treatments utilized for disease progression, n (%) ^a^	
Pain management	32 (42.7)
Surgical	23 (30.7)
MEK inhibitor^d^	18 (24.0)
Other	13 (17.3)
Pharmacotherapy	9 (12.0)
Conventional chemotherapy	4 (5.3)
Radiotherapy	2 (2.7)

Abbreviations: MEK, mitogen-activated protein kinase; MRI, magnetic resonance imaging; PET, positron emission tomography; PN, plexiform neurofibroma; SD, standard deviation.

Data in this table was collected in the period during which the patient was seen in the clinic during the study period, between the first visit and the last visit (prior to December 31, 2019)

a Multiple responses allowed, so rows may add up to greater than 100%.

b See Table S1 for other complications reported.

c See Table S1 for other symptoms reported.

d MEK inhibitors were not approved for treatment of PNs associated with NF1 during the study period but may have been used off-label or in clinical trials.

On average, 35.9% of patients developed ≥2 symptomatic, inoperable PNs that required treatment of any kind over the course of the study. The largest PNs most often occurred on the trunk (30.4%) and neck (13.7%). Over the course of the study, after the initial diagnosis of PNs, pain (76.5%), disfigurement (45.1%), and motor dysfunction (13.7%) were common complications of PNs. A full list of complications and symptoms can be found in Table S1. A very small number (2.9%) of patients had a malignancy develop (eg malignant peripheral nerve sheath tumor). Pain management was the most frequently reported intervention (45.2%) for the first symptomatic, inoperable PN diagnosed. Nearly 70% of patient medical records documenting progression of PNs reported pain as the most common indicator of progression. The most common methods used to detect the progression of PNs were MRI (85.3%) and clinical assessment without imaging (54.7%). Pain management (42.7%), surgery (30.7%), and MEK inhibitors (24.0%) were the primary interventions for progression, despite MEK inhibitor use being off-label during the study period.

### Treatment Characteristics

Treatment characteristics are presented in Table 4. Symptoms of NF1-PN were the most commonly cited reason for intervention (76.2%). Among patients who received treatment of any type during the study period (*n* = 84, 82.4%), 38.1% had at least 1 debulking or partial resection surgery performed, and nearly one-third (32.1%) had at least 1 surgical procedure of a different type. Patients underwent an average of 1.6 ± 0.9 unique surgeries, which targeted an average of 1.5 ± 2.0 unique PNs. Surgical intervention was most commonly performed on the trunk (28.1%) and extremities (28.1%), and 11.9% of patients underwent at least 1 spinal surgery. Nerve damage was reported as a complication in 15.6% of debulking surgeries or partial resections, and 3 patient records (9.4%) reported other complications.

**Table 4.** Treatment characteristics.

Characteristic	*n* (%)
Had treatment of any kind while a patient at the clinic, *n* (%)	84 (82.4)
Anatomical location of all symptomatic, inoperable PNs ever treated while a patient at the clinic, regardless of where the PN was first diagnosed (among patients who had treatment of any kind while a patient at the clinic), *n* (%) ^a^	
Trunk	30 (35.7)
Lower extremity	18 (21.4)
Other ^b^	15 (17.9)
Face	13 (15.5)
Neck	13 (15.5)
Upper extremity	8 (9.5)
Chest	7 (8.3)
Head	6 (7.1)
Eye	5 (6.0)
Intervention for the patient’s first symptomatic, inoperable PN treated (among patients who had treatment of any kind while a patient at the clinic), *n* (%) ^a^	
Pain management	38 (45.2)
Surgical	32 (38.1)
MEK inhibitor^c^	20 (23.8)
Other	10 (11.9)
Chemotherapy	6 (7.1)
Radiotherapy	2 (2.4)
Intervention trigger for the patient’s first symptomatic, inoperable PN treated at the clinic (among patients who had treatment of any kind while a patient at the clinic), *n* (%) ^a^	
Symptoms	64 (76.2)
Disfigurement	28 (33.3)
Risky location (eg head, neck, spine)	20 (23.8)
Mobility	12 (14.3)
Proximity to vital structures	11 (13.1)
Other	8 (9.5)
Had pharmacotherapy to treat ≥1 symptomatic, inoperable PNs (among patients who had treatment of any kind while a patient at the clinic), *n* (%)	53 (63.1)
Pharmacotherapy agent used	
Gabapentin	16 (30.2)
Selumetinib	13 (24.5)
Trametinib	9 (17.0)
NSAIDs	7 (13.2)
Other	17 (32.1)
Had ≥1 unique debulking or partial resection surgery performed on symptomatic, inoperable PNs of this patient while being seen at the clinic, *n* (%)	32 (38.1)
Among all PNs treated with debulking or partial resection surgery while at the clinic, how many were treated with medication, conventional chemotherapy, and/or radiotherapy to prevent regrowth following the surgery (among patients for whom data was reported, *n* (%)	31 (96.9)
Received surgical intervention other than debulking or partial resection	27 (32.1)
Spinal surgery	10 (11.9)
Reconstructive surgery	4 (4.8)
Other^ b^	16 (19.0)

Abbreviations: MEK, mitogen-activated protein kinase; NSAID, nonsteroidal anti-inflammatory drug; PN, plexiform neurofibroma.

Data in this table was collected in the period during which the patient was seen in the clinic during the study period, between the first visit and the last visit (prior to December 31, 2019).

a Multiple responses allowed, so rows may add up to greater than 100%.

b Other anatomical locations reported include abdomen, cervical plexus, every spinal nerve root, left axilla, left sciatic nerve, orbit, paraspinal, pelvis, right and left brachial plexus, scalp, and upper mediastinum.

c MEK inhibitors were not approved for treatment of PNs associated with NF1 during the study period but may have been used off-label or in clinical trials.

Among patients receiving treatment of any kind, pharmacotherapy was the most common nonsurgical intervention, with 63.1% of patients receiving pharmacotherapy during the study period and an average of 20.2 ± 18.0 months of use. The median age at initiation of pharmacotherapy was 10.5 years. Pharmacotherapies used included immune modulators, small molecule inhibitors, analgesics, anticonvulsants, and hypertensive medications. Among patients who received pharmacotherapy, gabapentin (30.2%), selumetinib (24.5%), and trametinib (17.0%) were the most common; pain medications included nonsteroidal anti-inflammatory drugs (13.2%), over-the-counter pain medication (7.5%), oxycodone (1.9%), cyclobenzaprine (1.9%), and 5% lidocaine patches (1.9%). Mitogen-activated protein kinase inhibitors were used as the intervention for the patient’s first symptomatic, inoperable PNs in 23.8% of cases. Furthermore, 96.9% of patients who underwent a debulking or partial resection received medication, conventional chemotherapy, and/or radiotherapy to prevent regrowth following surgery. Few patients were reportedly treated with conventional chemotherapy (7.1%), and even fewer received radiotherapy (2.4%). Of the patients who received chemotherapy, 50% progressed during the study period, despite treatment.

### Healthcare Resource Utilization

Healthcare resource utilization, including clinic visits, diagnostic scans, and emergency department visits, was high across patients with NF1-PN in this study (Table 5). On average, patients visited an outpatient care site 6.0 ± 6.2 times annually. At least 1 visit to specialists over the study period was common, including ophthalmologists (85.3%), oncologists (59.8%), neurologists (57.8%), and genetic counselors (53.9%). On average, patients attended 1.0 ± 2.2 oncologist visits, 0.9 ± 1.4 ophthalmology visits, and 0.5 ± 1.0 neurologist visits annually. Nearly 60% of the patient sample visited an emergency department at least once during the study observation period, with an average of 0.3 ± 0.5 emergency department visits per patient per year. Roughly half of the patients (49.0%) were admitted to the hospital at least once during the study period, with an average of 0.2 ± 0.4 hospitalizations per patient per year.

**Table 5.** Healthcare resource utilization.

	Patients who utilized healthcare resources (*N*=102), *n* (%)	No. of scans or visits among all patients (*N* = 102), mean ± SD	Annualized no. of scans or visits among all patients (*N* = 102), mean ± SD
Diagnostic scan			
Scan (any kind)	101 (99.0)	25.8 ± 27.2	3.4 ± 2.8
MRI scan	101 (99.0)	14.4 ± 14.1	2.0 ± 1.7
X-ray	82 (80.4)	10.3 ± 14.3	1.3 ± 1.5
CT scan	41 (40.2)	3.1 ± 4.0	0.4 ± 0.5
Ultrasound	37 (36.3)	3.2 ± 3.6	0.4 ± 0.6
PET scan	15 (14.7)	1.9 ± 1.4	0.2 ± 0.1
Whole body MRI scan	10 (9.8)	2.7 ± 3.2	0.3 ± 0.3
Hospitalization	50 (49.0)	1.5 ± 2.8	0.2 ± 0.4
Emergency department visit	61 (59.8)	2.0 ± 2.7	0.3 ± 0.5
Total outpatient care visit	101 (99.0)	40.9 ± 35.5	6.0 ± 6.2
Primary care visit	30 (29.4)	4.8 ± 11.6	0.6 ± 1.3
Oncologist visit	61 (59.8)	5.9 ± 9.4	1.0 ± 2.2
Radiation oncology visit	2 (2.0)	0.1 ± 0.6	0.0 ± 0.1
Ophthalmologist visit	87 (85.3)	5.8 ± 7.3	1.1 ± 1.4
Orthopedic surgeon visit	51 (50.0)	2.5 ± 4.3	0.6 ± 0.6
Behavioral health visit	41 (40.2)	1.8 ± 4.4	0.2 ± 0.6
Neurologist visit	59 (57.8)	3.1 ± 4.4	0.5 ± 1.0
Neuro-oncologist visit	30 (29.4)	4.8 ± 10.8	0.8 ± 1.9
Genetic counseling visit	55 (53.9)	1.6 ± 1.9	0.2 ± 0.2
Physical therapy visit	27 (26.5)	4.1 ± 13.2	0.6 ± 2.0

Abbreviations: CT, computerized tomography; MRI, magnetic resonance imaging; PET, positron emission tomography; SD, standard deviation.Healthcare resource utilization occurred while study subjects were a patient in the clinic, between the first visit and the last visit prior to December 31, 2019.

Nearly every patient received at least 1 diagnostic imaging scan over the study period (*n*=101, 99.0%), with an average of 3.4 ± 2.8 scans per patient per year (Table 5). Magnetic resonance imaging was the most frequently used diagnostic tool reported, with 99.0% of patients receiving at least 1 MRI in the study period and undergoing 2.0 ± 1.7 MRI scans per patient per year, on average.

The majority of patients (80.4%) received at least 1 x-ray, with an average of 1.3 ± 1.5 x-rays per patient per year, and 40.2% of patients received at least 1 CT scan, with an average of 0.4 ± 0.5 CT scans per patient per year among patients who received at least 1 CT.

## Discussion

To our knowledge, this is the first retrospective, noninterventional medical record abstraction study that describes the natural history and real-world burdens related to NF1-PN for patients and their families in the United States. From diagnosis, patients face disease progression with the potential for increasingly severe symptoms, varying and sometimes ineffective treatment approaches, and substantial HCRU that impact patients, their families, and healthcare systems. Nearly all patients in this study experienced at least 1 manifestation of NF1 beyond PN, and over two-thirds of patients experienced at least 1 morbidity or complication of NF1. All patients had at least 1 symptomatic, inoperable PN, with an average of almost 4 PNs per patient by the end of the study period. Pain and disfigurement were common complications of symptomatic PNs. Furthermore, patients underwent substantial monitoring to manage disease progression, including an average of 6 outpatient visits and 3 diagnostic imaging scans per year, posing a considerable burden to these patients and their families. The sizeable HCRU, a multitude of interventions, frequent disease progression, and variability of clinical events and outcomes all point to a substantial burden of illness associated with symptomatic, inoperable PNs in NF1 and the need for more effective treatment, as this study was conducted prior to the approval of MEK inhibitors. Despite NF1 manifestations being widespread and affecting many body systems, our findings on the most common clinical features (eg café-au-lait macules, intertriginous freckling, and Lisch nodules) and frequency of behavioral deficit and cognitive development impairment are in line with previous research.^4^^,^^21^ Our study also found that most patients received an initial diagnosis of NF1 and primary treatment from a geneticist, although genetic testing was an uncommon diagnostic method and most patients were diagnosed on the basis of the presence of café-au-lait macules or freckling in the axillary or inguinal regions. Diagnosis of PNs and primary treatments of PNs were administered by a variety of specialists across the study population, including pediatricians, neurologists, oncologists, dermatologists, and surgeons. The breadth of specialists involved in patient care demonstrates the heterogeneity in the diagnostic journey from patient to patient and illustrates a complex care environment for patients and their families.

This study demonstrated the burden of pain and disfigurement for patients with NF1, both of which were common complications of symptomatic PNs. Chronic pain can profoundly diminish quality of life,^22^^,^^23^ and disfigurement of any kind and on any part of the body can greatly impact identity, mental well-being, and social anxiety.^24–27^ Pain management was the most common treatment for PNs, according to both our study (45.2%) and a real-world study conducted by Yoshida et al^28^ in Japan. Many of the interventions available to patients during this study period were intended to treat symptoms (ie pain management) but did not address disease progression (eg shrinking tumors or halting or slowing tumor growth). The most common form of pain management was gabapentin, which is used for neuropathic pain.^29^ Among patients with at least 12 months of follow-up after surgical interventions, more than half of PNs regrew. Chemotherapy was not widely utilized and was not effective among patients in this study. The findings of this study, which was conducted prior to MEK inhibitor approvals, underscore the need for more effective and long-lasting treatment options.

Healthcare resource utilization also was high among patients with NF1-PN. On average, patients made 6 visits to an outpatient care center and 1 visit to an oncologist each year. Nearly all patients (99.0%) received at least 1 diagnostic or imaging scan, with 3.4 scans per year on average. A previous study by Yang et al^30^ in 2020 reported the mean total healthcare costs per NF1-PN patient per year in the United States as $38 292 ± $80 556, with outpatient care accounting for 67.5% of total cost. Almost half (49.0%) of our study population had at least 1 hospitalization reported, and 59.8% reported at least 1 visit to the emergency department, which is a higher proportion than that found in previous reports.^2^^,^^30^^,^^31^ The hospitalization rate documented here is almost 4 times greater than that of commercial insurance or Medicaid claim-based analyses reported for patients with NF1^32^ and PNs^30^ (approximately 13% in both instances). Beyond healthcare-related burdens, cognitive and behavioral difficulties have been documented in patients with NF1.^33^ Patients with NF1 are less likely to perform well in academic environments, and these academic difficulties get worse with age.^34^ This study reinforces the evidence that there are substantial burdens in the US population with NF1 and systematic, inoperable PNs and that these burdens likely have been underestimated in the past.

Nonetheless, this retrospective medical record review has certain inherent limitations. Information captured via the electronic case report form prepared for this study was limited to information available in the patients’ medical records. Additionally, data entered into the case report form may be subject to entry errors and resulting inaccuracies in reporting, although data-validation mechanisms minimized the risk of entry errors. Clinics in this study specialized in NF1 care, which may mean that the patients with more severe NF1 are overrepresented at these sites. These specialist centers may also explain the high percentage of patients receiving care from geneticists, despite genetic testing being an uncommon diagnostic method for NF1 in this study. Information on healthcare services received outside the site may not have been available for all patients. Furthermore, site interest in this study may have been limited during the COVID-19 pandemic due to competing priorities, potentially contributing to the smaller-than-desired number of participating sites. However, retrospective medical record abstraction of patient data provides a unique opportunity to collect and analyze real-world data outside the highly controlled setting of clinical trials, as well as provide richer clinical detail than typically may be available in existing administrative databases. Further, healthcare providers familiar with NF1 served as the direct data extractors, therefore minimizing chances of errors and accurate interpretation of notes and records. As indicated in a recent NF1-related systematic literature review, there is very little information on HCRU and costs associated with the care of NF1 and PNs.^3^ Our study further complements the published literature in this area by documenting HCRU across all payers for several distinct types of care, including emergency department visits, outpatient encounters, and hospitalizations. The HCRU data reported here suggest an additional economic burden. Future research that comprehensively investigates the economic burden associated with the HCRU of patients with NF1-PN is merited. Additionally, this study focused on inoperable PNs, which often are not differentiated from operable PNs in real-world studies, especially retrospective claims-based studies. Real-world data on patients who have received usual care may help to further contextualize findings from interventional studies. Finally, this study is one of the few that focus on burdens affecting patients with NF1-PN as well as their providers. It will be important to re-examine the natural history and real-world burdens of NF1 and associated PNs following the FDA approval of MEK inhibitors, given that any MEK inhibitor use in this study was off-label.

## Conclusion

This study highlights the high disease burden and the associated HCRU of patients with NF1-PN before pharmacologic treatments were available. Before MEK inhibitors were approved for the treatment of NF1-PN, treatment options were limited to symptom management and surgery primarily focused on reducing pain and disfiguration, though most interventions were largely ineffective and often carried significant risks (eg surgery). These findings reinforce the importance of effective, long-lasting treatments that address PN-associated symptoms, hinder disease progression, and lower HCRU.

## Supplementary Material

npag038_Supplementary_Data

## Data Availability

Alexion, AstraZeneca Rare Disease will consider requests for disclosure of clinical study participant-level data provided that participant privacy is assured through methods like data de-identification, pseudonymization, or anonymization (as required by applicable law), and if such disclosure was included in the relevant study informed consent form or similar documentation. Qualified academic investigators may request participant-level clinical data and supporting documents (statistical analysis plan and protocol) pertaining to Alexion-sponsored studies. Further details regarding data availability and instructions for requesting information are available in the Alexion Clinical Trials Disclosure and Transparency Policy at https://www.alexionclinicaltrialtransparency.com/data-requests/
