# Supplementary material for: Treatment patterns and healthcare resource utilization in pediatric neurofibromatosis type 1-associated symptomatic plexiform neurofibroma in the United States from 2000 to 2019
Source: Neurooncol Pract. 2026 Jun 13;13(5):1012–22. doi: 10.1093/nop/npag038 (PMC13578489; doi:10.1093/nop/npag038)
Supplement: Supplementary file 1 [file npag038_supplementary_data.docx]

# Supplemental Material

Table S1.	PN Complications and Symptoms Experienced With Disease Progression

| Other PN complications | Other symptoms |
| --- | --- |
| **GI related**  Abdominal pain and distention; GI malabsorption; difficulty with chewing; intermittent constipation; enuresis; encopresis; total parenteral nutrition  **Respiratory related**  Airway compression; airway compression and upper respiratory infections; breathing problems; decreased aeration of left lung; respiratory compromise; restrictive lung disease, dural ectasia, dyspnea; tracheostomy and ventilator dependence; recurrent pneumonias  **Mobility related**  Abnormal gait and itching; compromised mobility  **Skeletomuscular related**  Erosion of the mandibular ramus; tibial bowing; remodeling of bone; problems with chewing; scoliosis; erosion of bone  **Neurological related**  Burning sensation; dural ectasia and widening of lumbar spinal canal  **Speech, vision, or hearing related**  Difficulty with speech and articulation; speech difficulty; hearing loss; hearing loss and impaired vision; hypertension; hearing loss; swelling, proptosis of eye; ptosis  **Urinary related**  Urinary incontinence; urinary retention; renal artery stenosis; enuresis  **Cardiovascular related**  Right carotid compression; encasement of major vessels  **Other**  Itching; severe itching; loss of function (unspecified); precocious puberty; headache; swelling; infections; sleep apnea; inability to eat | **GI related**  GI issues; GI bleeding; duodenum compression (PN related); constipation  **Respiratory related**  Airway compression; airway issues; breathing problems (PN related); increased compression of the trachea; ventilator dependence; airway compression (PN related); atelectasis; aspiration  **Mobility related**  Mobility difficulties  **Skeletomuscular related**  Worsening scoliosis  **Neurological related**  Dural ectasia and widening of lumbar spinal canal; malignant peripheral nerve sheath tumor  **Speech, vision, or hearing related**  Speech/developmental delays; eyelid drooping and vision problems (PN related); optic glioma; hearing loss (PN related)  **Urinary related**  Urinary retention  **Cardiovascular related**  Heart murmur; hypertension; decreased cardiac ejection fraction  **Cognitive related**  Increase in anxiety; behavioral issues  **PN related**  Increase in number of PNs; increase in location and size of PNs; PN growth  **Other**  Dysphagia; short stature; tingling/itching (PN related); itching; delayed puberty; stinging sensation, itching; cutaneous neurofibromas; infections; swelling; leg length discrepancy; sleep problems; weakness |

GI = gastrointestinal; PN = plexiform neurofibroma.
